# Hsa_circ_0005325 Regulates the Proliferation, Apoptosis, Colony Formation, Migration, and Angiogenesis‐Promoting Behavior of Oral Squamous Cell Carcinoma Cells Through the miR‐433‐3p/HMGA2 Axis

**DOI:** 10.1002/cre2.70208

**Published:** 2025-09-03

**Authors:** Zhihan Lin, Yating Fu, Lei Mao, Hongjuan Yan, Wen Liu, Xiaoxue Tang

**Affiliations:** ^1^ Department of Stomatology, The First Affiliated Hospital, School of Medicine Shihezi University Shihezi China; ^2^ Department of Radiology of Urumqi Stomatological Hospital Urumqi Xinjiang China; ^3^ Department of Ultrasound The First Affiliated Hospital of Shihezi University Shihezi China

**Keywords:** CAL‐27 cells, hsa_circ_0005325, oral squamous cell carcinoma, SCC25 cells

## Abstract

**Objective:**

To explore the mechanism by which hsa_circ_0005325 affects the proliferation, apoptosis, colony formation, migration, and angiogenesis‐promoting behavior of oral squamous cell carcinoma cells through the miR‐433‐3p/HMGA2 axis.

**Material and Methods:**

qRT‒PCR was used to measure the expression of hsa_circ_0005325 in SCC25 and CAL‐27 cells and normal human oral epithelial cells (HOK). SCC25 and CAL‐27 cells were cultured, and Cell Counting Kit‐8 (CCK‐8), cell apoptosis, plate colony formation, Transwell migration and a tube formation assays were used to detect changes in cell proliferation, apoptosis, colony formation, migration and angiogenesis, respectively.

**Results:**

The expression of hsa_circ_0005325 was significantly increased in SCC25 and CAL‐27 cells. Compared with those in the sh‐NC group, the percentages of apoptotic SCC25 and CAL‐27 cells in the sh‐circ_0005325 group were significantly greater, and their proliferation, colony formation, migration and angiogenesis capacities were significantly lower (*p* < 0.05). Moreover, the protein expression level of HMGA2 was significantly decreased, and the expression level of miR‐433‐3p was significantly increased in the sh‐circ_0005325 group versus the control group (*p* < 0.05).

**Conclusion:**

Hsa_circ_0005325 is highly expressed in SCC25 and CAL‐27 cells. Downregulation of hsa_circ_0005325 can inhibit the proliferation and invasion of SCC25 and CAL‐27 cells and promote their apoptosis.

## 1 Background

Oral squamous cell carcinoma (OSCC) is a common malignant tumor in stomatology. It has a high prevalence and a high incidence worldwide and seriously affects the survival times of patients (Nagao and Warnakulasuriya 2020). Circular RNAs (circRNAs) constitute a newly discovered class of noncoding RNAs and have been reported to be involved in the progression of various diseases, including cancer (Wu et al. 2023). In recent years, increasing evidence has shown that circRNAs, such as those related to cell viability, differentiation, and apoptosis, are involved in the pathogenesis of tumors (Chen et al. 2022). Studies have shown that hsa_circ_0072387 inhibits the progression of OSCC by downregulating miR‐503‐5p (Han et al. 2021). Through high‐throughput sequencing of circRNAs, several researchers have found that hsa_circ_0005325 is highly expressed in OSCC tumor tissues (Xu et al. 2021). However, there remains a significant gap in current research on OSCC‐related circRNAs: most studies only focus on the expression differences and preliminary functional verification of a single circRNAs, and the analysis of their specific regulatory networks, such as the molecular mechanism of acting as competing endogenous RNA (ceRNA) sponges to adsorb microRNAs (miRNAs), is not systematic. In particular, there is a lack of in‐depth exploration of the synergistic role of the “circRNA‐miRNA‐target gene” axis in OSCC malignant phenotypes, such as angiogenesis and epithelial‐mesenchymal transition. The main objective of this study was to explore the effects of hsa_circ_0005325 via the miR‐433‐3p/HMGA2 axis on the proliferation, migration, invasion, and lumen formation of OSCC cells in OSCC progression, as well as the underlying mechanism, to provide ideas for the clinical treatment of OSCC.

## 2 Materials and Methods

### 2.1 Main Materials

The human OSCC cell lines SCC25 and CAL27 and the normal human oral epithelial cell line HOK were purchased from Wuhan Procell Life Technology Co. Ltd. An Annexin V‐FITC/PI kit was purchased from BD Biosciences (USA) for flow cytometry experiments. The transfection reagent Lipofectamine 3000 and the RNA extraction reagent TRIzol (used for processing before qRT‒PCR) were purchased from Invitrogen (USA). RNase‐free centrifuge tubes were purchased from Axygen (USA). DEPC water, Cell Counting Kit‐8 (CCK‐8) reagents, electrophoresis buffer (Tris‐Gly, powder, in bags), an enhanced chemiluminescence (ECL) chemiluminescence kit, rapid membrane transfer solution, a protein molecular weight marker, and Matrigel were purchased from Shanghai Beyotime Institute of Biotechnology Co. Ltd. The RIP‐related reagent PureBinding RNA Immunoprecipitation Kit was purchased from Guangzhou GeneScience Biotechnology Co. Ltd. The target protein/IgG antibody (IP) for RIP experiments and the primary antibody for Western blotting (WB) experiments were purchased from Wuhan Sanying Biotechnology Co. Ltd. The SDS‒PAGE precast gel (10%) for WB experiments was purchased from Yeasen Biotechnology (Shanghai) Co. Ltd. The Tris‐buffered saline containing Tween 20 (TBST) and antibody dilution buffers for WB experiments were purchased from Beijing Solarbio Science & Technology Co. Ltd. The PVDF membrane for WB experiments was purchased from Millipore, USA. Matrigel was purchased from BD, USA, and Transwell chambers were purchased from Corning, USA. The PCR primers used were synthesized by Shanghai Sangon Biotech Co. Ltd. miR‐433‐3p mimic, miR‐433‐3p mimic NC, miR‐433‐3p inhibitor, and miR‐433‐3p inhibitor NC were purchased from Suzhou GenePharma Co. Ltd.

## 3 Methods

### 3.1 Cell Culture and Transfection

The human OSCC cell lines CAL‐27 and SCC25 were cultured in DMEM containing 10% fetal bovine serum, penicillin (100 U/mL), and streptomycin (100 µg/mL) in an incubator at 37°C with 5% CO_2_. The cells were divided into sh‐NC and sh‐hsa_circ_0005325 groups for experiments. Cell growth was observed. When the cell confluence reached approximately 70%, the sh_circ_0005325 plasmid or negative control plasmid (sh‐NC) was transfected into the cells using Lipofectamine 3000 transfection reagent. After 6 h of transfection, the medium was replaced with complete medium, and the cells were returned to the incubator for an additional 48 h of culture before subsequent experiments.

### 3.2 Database Analysis

The circBase (http://www.circbase.org/) and CircInteractome (http://circinteractome.nia.nih.gov/) databases and tools were used to predict and analyze HMGA2‐related circRNA molecules. The starBase (http://starbase.sysu.edu.cn/in) online database was used to predict the downstream target genes of miR‐433‐3p in OSCC cells.

### 3.3 Flow Cytometry

For flow cytometry, the transfected cells were obtained after washing with PBS and then resuspended and stained using the method recommended by the manufacturer of the Annexin V‐FITC/PI kit. The percentage of apoptotic cells in each group was subsequently detected by flow cytometry within 30 min. Flow cytometry assays for apoptosis were performed in three independent biological replicates (*n* = 3), with each replicate analyzing ≥ 10,000 cells per group.

### 3.4 Quantitative Real‑Time Polymerase Chain Reaction (qRT‑PCR)

Total RNA was extracted from the cells via a total RNA extraction kit. The RNA was reverse‐transcribed into cDNA using the AMV First‐Strand cDNA Synthesis and miRNA First‐Strand cDNA Synthesis kits. The sequences of primers used were as follows: for miR‐433‐3p, the upstream primer was 5′‐GCGATCATGATGGGCTCCT‐3′, and the downstream primer was 5′‐AGTGCAGGGTCCGAGGTATT‐3′; for U6, the upstream primer was 5′‐CTCGCTTCGGCAGCACA‐3′, and the downstream primer was 5′‐AACGCTTCACGAATTTGCGT‐3′. The reaction conditions were as follows: 50°C for 2 min, predenaturation at 95°C for 2 min, 95°C for 15 s, and 60°C for 1 min for 40 cycles. Using U6 as an internal reference, qRT‒PCR was performed using SYBR Green Master Mix, and the relative expression level of cellular RNA was calculated using the $2^{-∆∆C_{T}}$ method.

### 3.5 CCK‐8 Assay

Cell viability was evaluated via CCK‐8 assays. The cell concentration was adjusted to 2 × 10^5^/mL, and the cells were seeded into 96‐well plates at a density of 3000 cells per well. The proliferation ability of cells cultured for 0, 24, 48, or 72 h was assessed. Each well was washed twice with PBS, and then, serum‐free medium containing 10 µL of CCK‐8 solution was added and incubated in the incubator in the dark for 1 h. The absorbance was measured at a wavelength of 450 nm using a microplate reader.

### 3.6 Plate Colony Formation Assay

The cells in each group (500 cells per well) were cultured in 24‐well plates for 10‒14 days. The cells were fixed with 4% paraformaldehyde for 30 min and stained with 0.2% crystal violet for 30 min. The number of colonies was quantified using the Image‐Pro Plus software.

### 3.7 Rna Binding Protein Immunoprecipitation (RIP)

The experiment was carried out using the Magna RIP RNA‐Binding Protein Immunoprecipitation Kit and an anti‐AGO2 antibody; the samples were divided into three groups: Input, anti‐IgG, and anti‐AGO2. The experimental steps included collection of the cell lysate, preparation of immunoprecipitated protein magnetic beads, RNA‐binding protein immunoprecipitation, RNA purification (including treatment with proteinase K buffer, phenol‒chloroform‐isopropanol extraction, and precipitation with absolute ethanol), and finally, detection of hsa_circ_0005325 enrichment via qRT‒PCR.

### 3.8 WB

Total cellular proteins were extracted using a protein extraction kit. After the protein samples were quantified via a bicinchoninic acid (BCA) protein quantitation assay kit, sodium dodecyl sulfate‒polyacrylamide gel electrophoresis (SDS‒PAGE) analysis was performed. The proteins were subsequently transferred to a polyvinylidene fluoride (PVDF) membrane. The PVDF membrane was blocked with 5% skim milk on a shaker at room temperature for 1 h. Subsequently, antibodies against HMGA2, E‐cadherin, Vimentin, Slug, Twist, VEGFA, VEGFC, FGF2, and the internal reference protein GAPDH were added and incubated overnight at 4°C. After the samples were washed with TBST, horseradish peroxidase‐labeled secondary antibody was added, and the samples were incubated for 2 h. Protein expression was detected using an ECL kit and a gel imaging system. Western blot experiments were repeated three times (*n* = 3 independent experiments), with each replicate including three technical repeats. Band intensities were quantified using ImageJ, and relative expression was normalized to GAPDH.

### 3.9 Dual‐Luciferase Reporter Assay

On the basis of the binding sites of hsa_circ_0005325 with miR‐433‐3p and miR‐433‐3p with the HMGA2 3′UTR, the fragments containing the putative (WT) and mutant (MUT) binding sites of miR‐433‐3p with the hsa_circ_0005325 or HMGA2 3′UTR were cloned and inserted into the pmirGLO luciferase reporter vector, namely, WT‐hsa_circ_0005325, WT‐HMGA2‐3′UTR, MUT‐hsa_circ_0005325, and MUT‐HMGA2‐3′UTR. To determine the binding strength of miR‐433‐3p with hsa_circ_0005325 or HMGA2, the above vectors were cotransfected with miR‐433‐3p or miR‐NC into OSCC cells. After 48 h, the luciferase activity was analyzed using a Dual‐Luciferase Reporter Assay System.

### 3.10 Tube Formation Assay

One day before the experiment, Matrigel was mixed with pure medium at a ratio of 1:3, and 50 µL per well was added to a 96‐well plate for precoating. The entire process was carried out on ice. OSCC cells in each group were cultured in serum‐free medium for 24 h, after which the supernatants were collected. The supernatant obtained by centrifugation was used to resuspend HUVECs to obtain a cell suspension. The cell suspension was seeded at a density of 2 × 10^4^ cells per well into a 96‐well plate precoated with Matrigel. After a 4‐h incubation in the incubator, tube formation was observed and photographed. The circumference of the tubes was measured using the Wimasis software, and the circumference of the tubes was used as the detection index. Tube formation assays were repeated four times (*n* = 4), with each experiment including three technical repeats (different fields of view per well).

### 3.11 Transwell Migration Assay

A total of 1–5 × 10^4^ cells were resuspended in 200 µL of serum‐free medium, and the transfected cell suspension was added to the upper chamber of the Transwell insert. In addition, the culture medium containing serum was added to the lower chamber of the 24‐well plate. After 24 h of culture, the nonmigrated cells on the upper surface of the membrane were gently removed with a cotton swab. The cells that invaded the lower surface of the upper chamber were fixed with 4% paraformaldehyde, stained with 1% crystal violet for 30 min, and then washed with ddH₂O. Finally, the number of invaded cells in five randomly selected visual fields was counted under a microscope, and the cells that passed through were quantitatively analyzed via ImageJ software. Three replicates were set for each group, and the average value was taken.

### 3.12 Statistical Analysis

The data were statistically analyzed using SPSS 27.0 software, and the images were generated via GraphPad 10 software. Independent‐samples *t* tests were used for intergroup comparisons between two groups, such as comparisons between the sh‐NC group and the sh‐hsa_circ_0005325 group. One‐way analysis of variance was used for intergroup comparisons among multiple groups, such as comparisons among the sh‐NC group, sh‐hsa_circ_0005325 group, sh‐hsa_circ_0005325 + anti‐miR‐433‐3p group, and sh‐hsa_circ_0005325 + oe‐HMGA2 group, and the least significant difference (LSD) test was used for pairwise intergroup comparisons among these multiple groups. The *χ* ^2^ test was used for counting data. *p* < 0.05 indicated a statistically significant difference.

## 4 Results

### 4.1 Hsa_circ_0005325 Expression Increases Significantly in Cancer Tissues and Inhibits Apoptosis in OSCC Tumor Tissues

**Figure 1:**
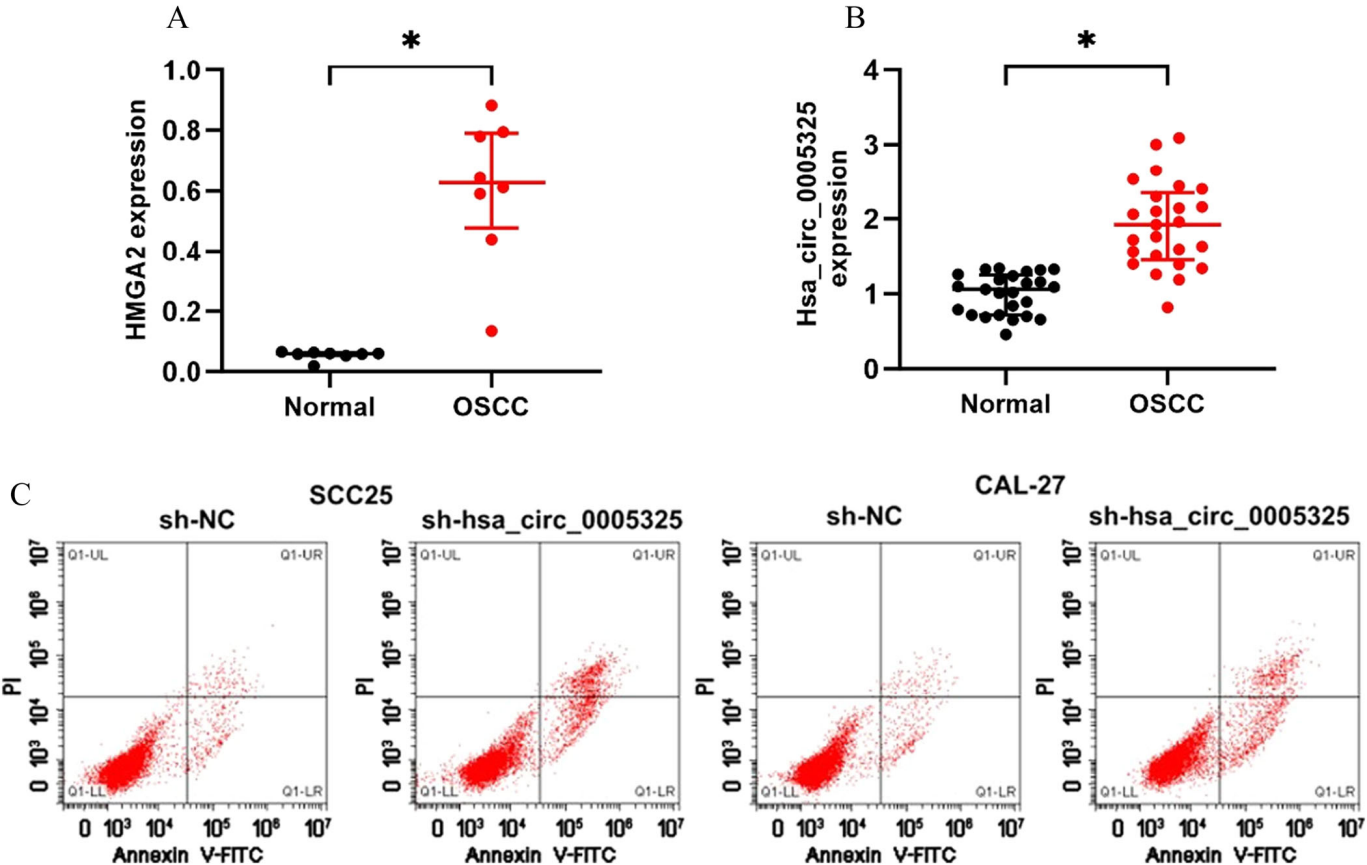
Expression of hsa_circ_0005325 in oral squamous cell carcinoma (OSCC) tumor tissues and normal tissues, as well as effects of its knockdown on apoptosis of OSCC tumor cells detected by flow cytometry. (A) HMGA2 protein expression in OSCC versus normal tissues (WB). (B) Hsa_circ_0005325 expression in OSCC tissues and cell lines (qPCR). (C) Effects of hsa_circ_0005325 knockdown on the apoptosis of OSCC tumor cells according to flow cytometry.

Western blot analysis revealed that HMGA2 protein expression was significantly upregulated in OSCC tumor tissues compared to normal tissues. (Figure 1A) Analysis using the circBase database revealed that two circRNA molecules were derived from the HMGA2 gene. The expression level of hsa_circ_0005325 in SCC25 and CAL‐27 cells was measured via qRT‒PCR. Compared with that in paired normal tissues, the expression of hsa_circ_0005325 was significantly increased in OSCC tumor tissues (Figure 1B).

Cell apoptosis was detected by flow cytometry. The results showed that after the expression of hsa_circ_0005325 was silenced in SCC25 and CAL‐27 cells, the percentage of apoptotic cells increased significantly (Figure 1C).

### 4.2 Hsa_circ_0005325 Promotes the Proliferation, Colony Formation, and Migration of OSCC Cells in Culture and in Tumor Tissues

**Figure 2:**
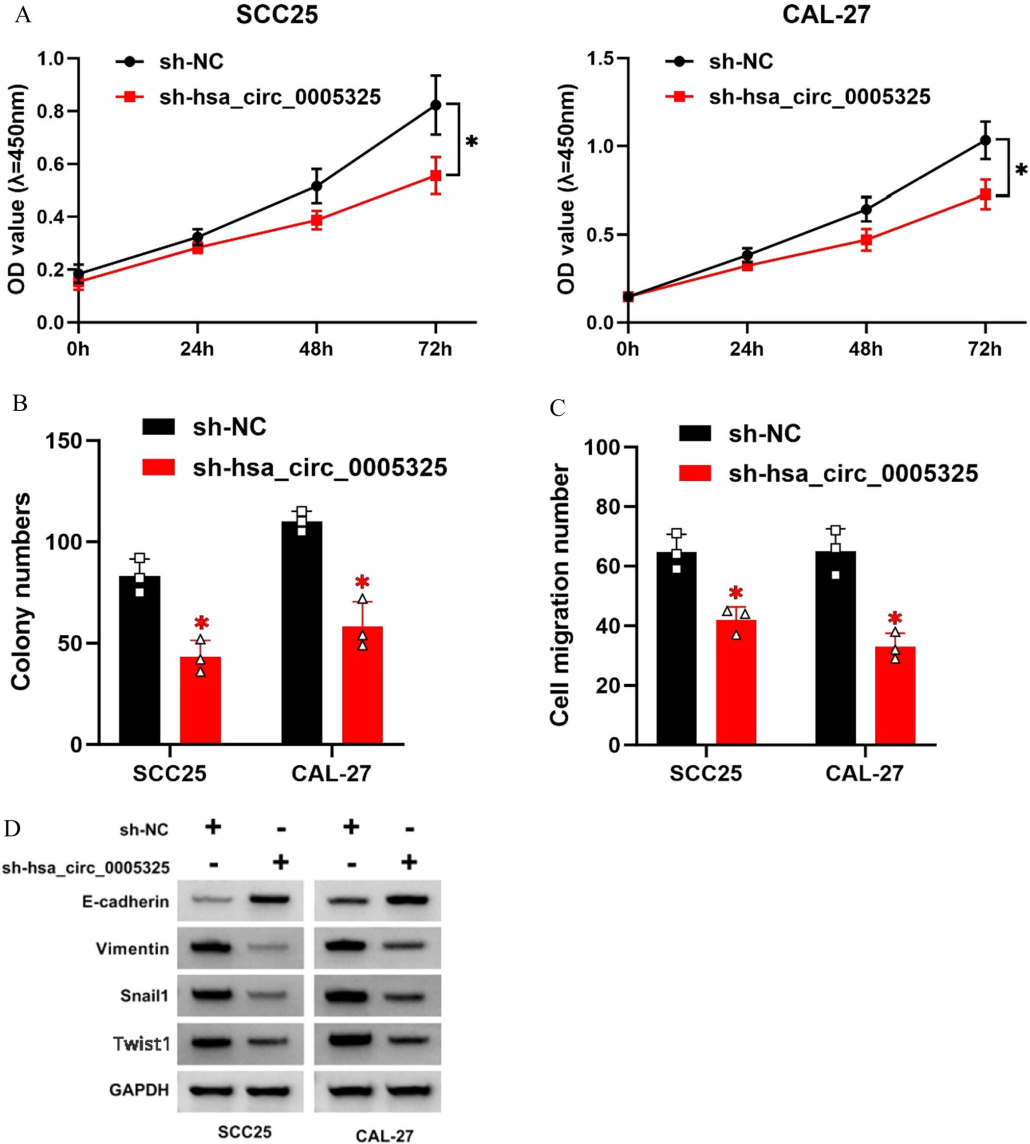
Effects of hsa_circ_0005325 knockdown on the biological behaviors of OSCC tumor cells. (A) Effects of hsa_circ_0005325 knockdown on the proliferation of OSCC tumor cells. (B) Effects of hsa_circ_0005325 knockdown on the colony formation of OSCC tumor cells. (C) Effects of hsa_circ_0005325 knockdown on the migration of OSCC tumor cells. (D) Western blot analysis of EMT‐related proteins: E‐cadherin, Vimentin, Snail1, and Twist1. GAPDH was used as an internal control. Each dot represents an individual tissue sample, and the bar indicates the mean value with standard deviation. *p < 0.05.

The role of hsa_circ_0005325 in the occurrence and metastasis of OSCC was investigated by knocking down hsa_circ_0005325 in OSCC cells. The results showed that knocking down hsa_circ_0005325 in SCC25 and CAL‐27 cells inhibited cell proliferation (Figure 2A), colony formation (Figure 2B), and migration (Figure 2C). In addition, it inhibited the expression of the epithelial marker E‐cadherin, promoted the expression of the mesenchymal marker vimentin, and increased the expression of the EMT (Epithelial‐Mesenchymal Transition)‐related transcription factors Snail1 and Twist1 (Figure 2D).

### 4.3 Hsa_circ_0005325 Exerts Its Effects by Sponging Mir‐433‐3P

**Figure 3:**
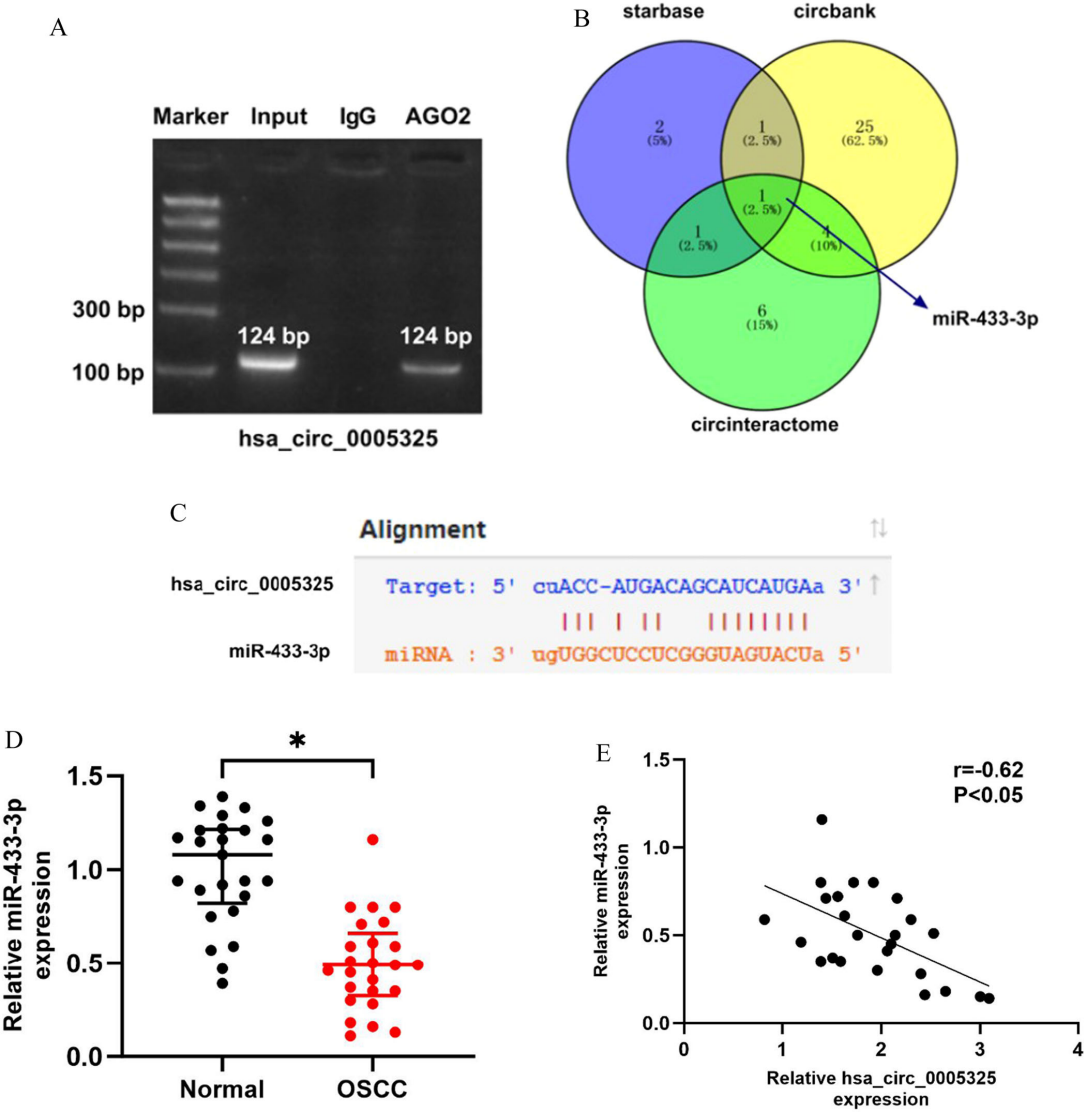
Preliminary study on the targeting relationship between miR‐433‐3p and hsa_circ_0005325. (A) Detection of the targeting relationship between hsa_circ_0005325 and AGO2 by AGO2‐RIP experiments. (B) Schematic diagram of the overlapping miR‐433‐3p predicted by starBase, CircBank, and CircInteractome. (C) Schematic diagram of the binding site between hsa_circ_0005325 and miR‐433‐3p. (D) Expression of miR‐433‐3p in normal tissues and OSCC tumor tissues, (E) Correlation between miR‐433‐3p expression and hsa_circ_0005325 expression in OSCC tumor tissues. *p < 0.05.

Previous studies have shown that hsa_circ_0005325 is expressed mostly in the cytoplasm of SCC25 and CAL‐27 cells. Through the AGO2‐RIP experiment, we found that hsa_circ_0005325 can bind to the AGO2 protein (Figure 3A), suggesting that hsa_circ_0005325 regulates the development and progression of OSCC by sponging miRNAs. Bioinformatics software, such as starBase, CircInteractome, and CircBank, were used for the prediction of miRNA targets and revealed that there are binding sites for miR‐433‐3p on hsa_circ_0005325 (Figure 3B,C). The results of qRT‒PCR revealed that the expression of miR‐433‐3p was downregulated in OSCC tumor tissues compared to normal tissues, and there was a negative correlation between the expression of miR‐433‐3p and that of hsa_circ_0005325 in tumor tissues (Figure 3D,E).

### 4.4 HMGA2 Is a Downstream Target Gene of mir‐433‐3P

**Figure 4:**
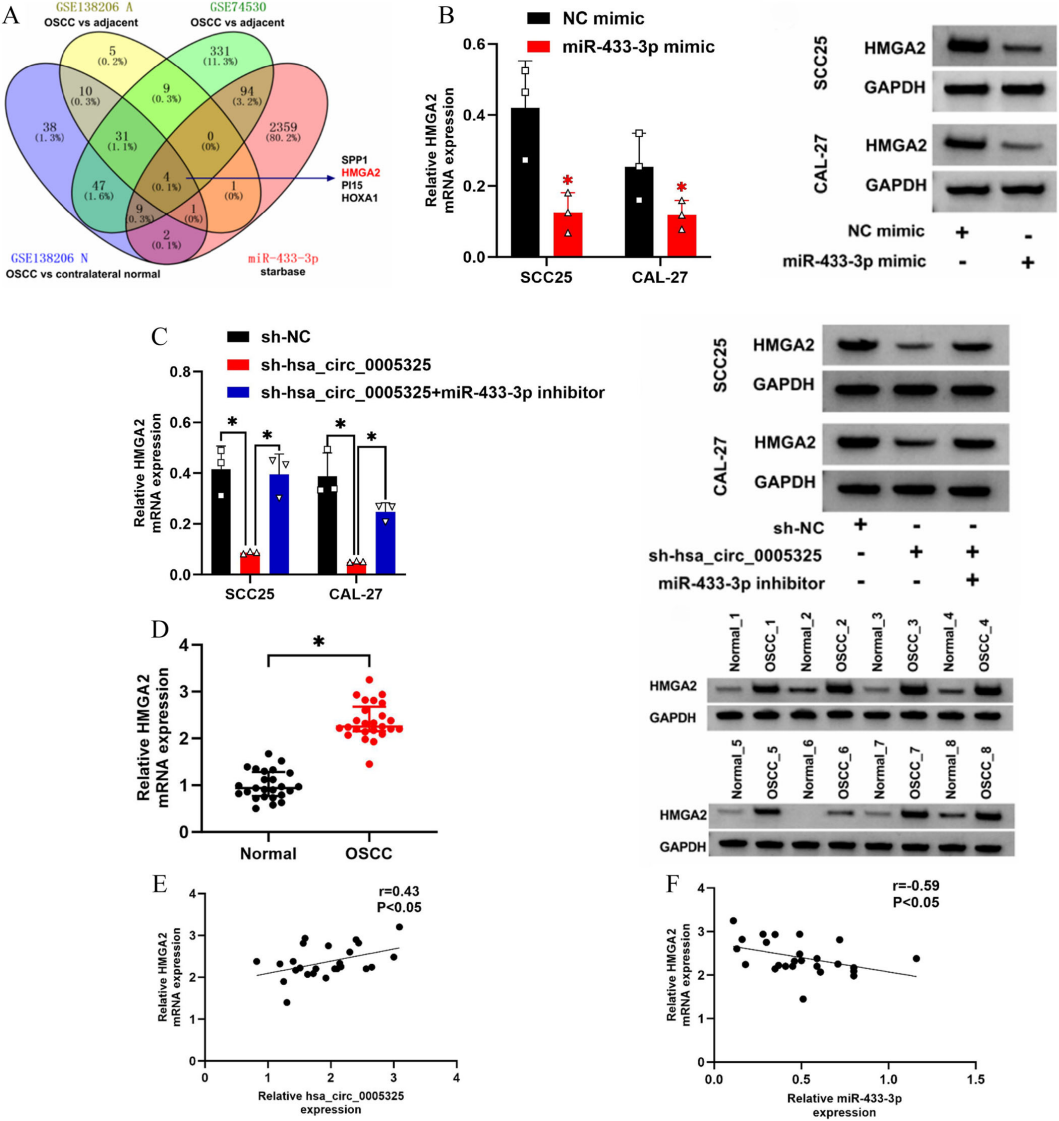
Preliminary study on the targeting relationship between miR‐433‐3p and HMGA2. (A) Schematic diagram of the overlapping HMGA2 genes predicted by GSE138206 A, GSE74530, GSE138206 N, and starBase. (B) Detection of the relationship between the expression of HMGA2 and miR‐433‐3p in OSCC tumor cells by Western blotting (WB). (C) Detection of the effects of the expression levels of hsa_circ_0005325 and miR‐433‐3p on the expression level of HMGA2 in OSCC tumor cells by WB. (D) Expression of HMGA2 in normal tissues and OSCC tumor tissues. (E) Relationship between the expression of HMGA2 and the expression of hsa_circ_0005325 in OSCC tumor tissues. (F) Relationship between the expression of HMGA2 and the expression of miR‐433‐3p in OSCC tumor tissues. Each dot represents an individual tissue sample, and the bar indicates the mean value with standard deviation. *p < 0.05.

In the GSE138206 data set, 61 genes were upregulated in OSCC compared with paracancerous tissues, and 142 genes were upregulated in OSCC compared with contralateral normal tissues. In the GSE74530 data set, 525 genes whose expression was upregulated in OSCC tissues compared with paracancerous tissues were identified. StarBase was used to predict that there were 2470 downstream target genes of miR‐433‐3p in OSCC cells. The results revealed that HMGA2 has potential binding sites for miR‐433‐3p (Figure 4A). The results of the qRT‒PCR and Western blot experiments revealed that the overexpression of miR‐433‐3p inhibited the expression of HMGA2 (Figure 4B) and that the overexpression of hsa_circ_0005325 attenuated the inhibitory effect of miR‐433‐3p on HMGA2 expression (Figure 4C). In addition, the expression of HMGA2 was upregulated in OSCC tumor tissues (Figure 4D). Moreover, there was a positive correlation between the expression levels of HMGA2 and hsa_circ_0005325 and a negative correlation between the expression levels of HMGA2 and miR‐433‐3p in OSCC tumor tissues (Figure 4E,F).

### 4.5 Hsa_circ_0005325 Promotes the Aggressive Behaviors of OSCC Cells by Regulating the miR‐433‐3p/HMGA2 Axis

**Figure 5:**
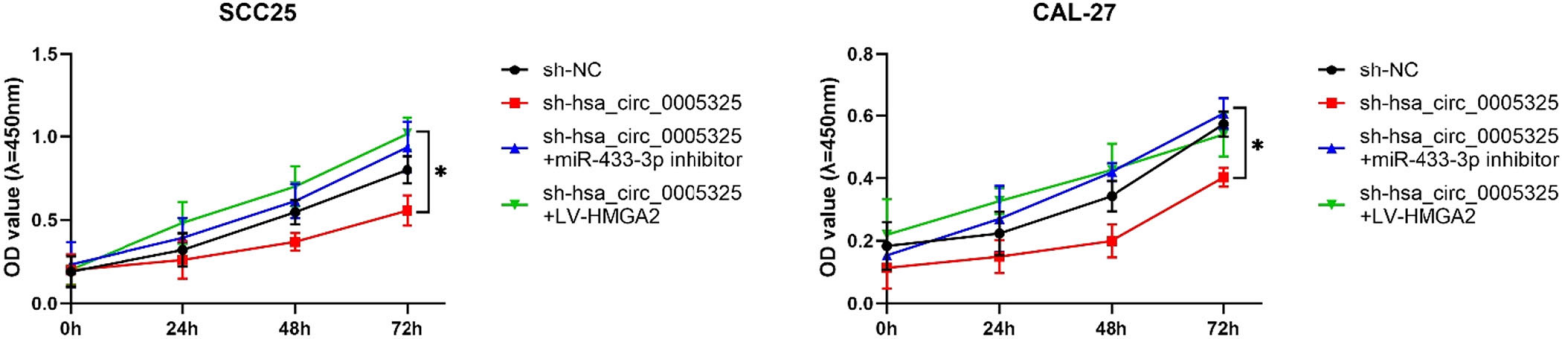
Detection of the relationships among hsa_circ_0005325, miR‐433‐3p, and HMGA2 expression levels in OSCC tumor tissues according to results of colony formation assays. *p < 0.05.

To verify the role of hsa_circ_0005325 in regulating the miR‐433‐3p/HMGA2 axis, SCC25 and CAL‐27 cells were transfected with sh‐NC, sh‐hsa_circ_0005325, sh‐hsa_circ_0005325 + anti‐miR‐433‐3p, or sh‐hsa_circ_0005325 + oe‐HMGA2. The data revealed that inhibiting the expression of hsa_circ_0005325 suppressed cell proliferation, whereas inhibiting miR‐433‐3p or overexpressing HMGA2 eliminated this inhibitory effect (Figure 5).

### 4.6 Hsa_circ_0005325 Promotes OSCC Cell‐Mediated Angiogenesis

**Figure 6:**
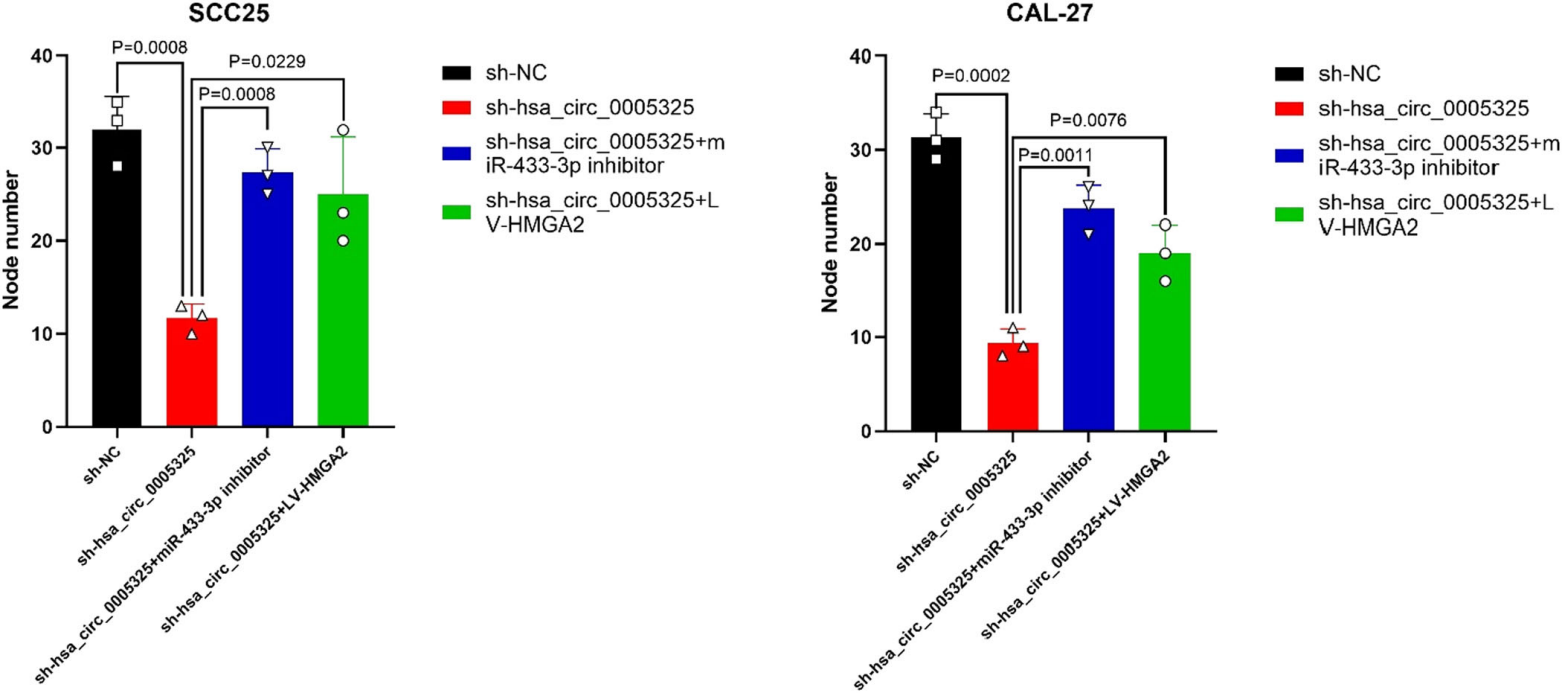
Detection of the effects of the expression levels of hsa_circ_0005325, miR‐433‐3p, and HMGA2 on the tube‐forming ability of HUVECs. ***p < 0.001, **p < 0.01, *p < 0.05. SCC25: ***p < 0.001 (sh‐circ vs. sh‐NC and sh‐circ+inhibitor vs. sh‐circ); *p < 0.05 (sh‐circ+LV‐HMGA2 vs sh‐circ). CAL‐27: ***p < 0.001 (sh‐circ vs. sh‐NC); **p < 0.01 (sh‐circ+inhibitor vs. sh‐circ and sh‐circ+LV‐HMGA2 vs. sh‐circ). Each dot represents an individual tissue sample, and the bar indicates the mean value with standard deviation.

The results of the in vitro angiogenesis assay revealed that, in SCC25 and CAL‐27 cells, compared with that in the sh‐NC group, the number of tubes formed in the sh‐hsa_circ_0005325 group decreased by 49.25% and 74.16% in SCC25 and CAL‐27 cells, respectively (*p *< 0.05). Compared with that in the sh‐hsa_circ_0005325 group, the number of tubes formed in the sh‐hsa_circ_0005325+miR‐433‐3p inhibitor group increased by 134.29% and 153.57% in SCC25 and CAL‐27 cells, respectively (*p *< 0.05). Compared with that in the sh‐hsa_circ_0005325 group, the number of tubes formed in the sh‐hsa_circ_0005325 + LV‐HMGA2 group increased by 114.29% and 103.57% in SCC25 and CAL‐27 cells, respectively (*p *< 0.05) (Figure 6).

## 5 Discussion

OSCC is one of the most common malignant tumors in the head and neck region. Clinically, it often involves regional lymph node metastasis, which impairs a patient's appearance, pronunciation, swallowing, and taste; thus, OSCC poses a serious threat to human health (Tan et al. 2023). Therefore, further clarification of the biological markers and therapeutic targets of OSCC is of clinical importance for OSCC diagnosis, treatment, and prognosis evaluation. In recent years, circRNAs have emerged as a major class of noncoding RNA molecules, many of which play important roles in the development and progression of cancer cells through different mechanisms (Kristensen et al. 2022). Research has revealed that circRNAs can exert their biological functions as microRNA sponges, RNA‐binding protein sponges, gene splicing and transcription regulators, and protein/peptide translators (Zhang, Luo et al. 2023). For example, the overexpression of hsa_circ_0060927 promotes the proliferation and migration of leukemia cells and inhibits apoptosis (Xu et al. 2021). Some studies have shown that circRNAs, as ceRNAs, regulate various signaling pathways to modulate lung cancer metastasis (Li et al. 2025). In addition, circRNAs can serve as diagnostic markers and therapeutic targets for gliomas or other diseases (Peng et al. 2022). Therefore, analyzing the role and mechanism of circRNAs in OSCC, with a focus on their application in the diagnosis and targeted therapy of OSCC, may reveal new clinical treatment options.

Hsa_circ_0005325 is derived from the human genome chr19:34921480‐34925873+ and is 321 nt in length. It is formed via circularization of exons 2, 3, 4, and 5 of the UBA2 gene (Zhong and Feng 2022; Glažar et al. 2014). Some researchers discovered through circRNA high‐throughput sequencing that circ_0005325 is highly expressed in OSCC tumor tissues (Xu et al. 2021), suggesting that circ_0005325 may be involved in the tumorigenesis of OSCC. We subsequently investigated the expression of circ_0005325 in clinical samples and reported that circ_0005325 is highly expressed in clinical OSCC tissues and cells. Functionally, circ_0005325 silencing inhibited the invasion and migration abilities of cancer cells but promoted their apoptosis. More importantly, knockdown of circ_0005325 suppressed the proliferation of OSCC tumor cells. In summary, these results demonstrate that circ_0005325 has an oncogenic effect; specifically, it promotes the development of OSCC.

Previous studies have shown that circRNAs can act as endogenous sponges for miRNAs, preventing the miRNA‐mediated degradation of target mRNAs (Zhang, Wang et al. 2023). Therefore, we hypothesized that circ_0005325 may also function by acting as a miRNA sponge. In this study, we confirmed that circ_0005325 directly binds to miR‐433‐3p to exert its effects.

Some studies in many human cancers have indicated that miR‐433‐3p acts as a tumor suppressor. For example, miR‐433‐3p inhibits the biological functions and malignant progression of gliomas by targeting and inhibiting the expression of AJUBA (Zhang et al. 2022). Experimental research has shown that knockout of circ_0001535 inhibits the growth of colorectal cancer by upregulating the expression of miR‐433‐3p and downregulating the expression of recombination signal‐binding protein Jκ(RBPJ) (Mao et al. 2023). The results of this study revealed that the expression of miR‐433‐3p is decreased in OSCC tissues versus normal controls and that the inhibition of miR‐433‐3p promotes the proliferation of OSCC cells, which is consistent with the findings of previous studies (Yang, Zhang et al. 2023). In addition, as expected, the inhibition of miR‐433‐3p was shown to reverse the inhibitory effect of circ_0005325 knockdown on the malignant biological behavior of OSCC cells. Therefore, we conclude that circ_0005325 promotes the development of OSCC by sponging miR‐433‐3p.

High‐mobility group protein A2 (HMGA2) is a nonhistone chromosomal protein that can regulate transcription by altering chromatin structure. Multiple studies have shown that the overexpression of HMGA2 can drive tumor development or promote the invasiveness of tumors (Wei 2022; Wang et al. 2022; Ma et al. 2024). In recent years, an increasing number of studies have demonstrated that the overexpression of HMGA2 is associated with lymph node metastasis, distant metastasis, and poor outcomes in OSCC patients (Gao et al. 2021; Liu et al. 2021; Yang, Liu et al. 2023). Moreover, some studies have confirmed that HMGA2 can promote the EMT of OSCC cells by regulating the expression of EMT markers such as E‐cadherin and vimentin and the transcription factors Slug and Twist. It can also regulate the expression of angiogenesis‐related genes, including vascular endothelial growth factor A (VEGFA), vascular endothelial growth factor C (VEGFC), and fibroblast growth factor 2 (FGF2), to promote angiogenesis, thereby facilitating the development and progression of OSCC (Liu et al. 2021; Li et al. 2022; Sakata et al. 2019). In this study, we confirmed that miR‐433‐3p directly targets HMGA2 and that circ_0005325 acts as a sponge for miR‐433‐3p to promotes the expression of HMGA2 in OSCC. In addition, overexpression of HMGA2 attenuated the antitumor effect of miR‐433‐3p overexpression on OSCC cells.

Research has shown that angiogenesis plays a crucial role in the growth, invasion, and metastasis of tumors, providing oxygen and the necessary nutrients for tumor cells (Liu et al. 2023). CircRNAs have been shown to be associated with angiogenesis (Ghaedrahmati et al. 2023), and some experimental studies have demonstrated their function in promoting angiogenesis (Razavi et al. 2021). For example, hsa_circ_0000520 inhibits the invasion, migration, and angiogenesis of bladder cancer cells by suppressing the Lin 28 a/PTEN/PI 3 K/AKT axis (Zhang et al. 2024). Research has established VEGFA as a pivotal regulator of angiogenesis. The molecular mechanism involves VEGFA binding to its cognate receptors (VEGFR1 and VEGFR2) on endothelial cell surfaces, thereby initiating downstream signaling cascades that stimulate endothelial cell proliferation, enhance cellular survival, and increase vascular permeability‐ultimately driving neovascularization (Zhang, Zhang et al. 2023). Furthermore, experimental evidence demonstrates a functional interplay between HMGA2 and VEGFA expression: HMGA2 knockdown significantly downregulates VEGFA levels, whereas HMGA2 overexpression conversely upregulates VEGFA expression (Yan et al. 2022). Sakata et al.'s study also demonstrated that in OSCC cell lines, after knocking down HMGA2, the mRNA expressions of VEGFA, VEGFC, and FGF2 were significantly downregulated, indicating that HMGA2 can positively regulate the transcription of these key angiogenesis‐related genes (Sakata et al. 2019). In this study, when the expression of hsa_circ_0005325 was inhibited in OSCC tumor tissues, compared with that in the sh‐NC group, the number of tubes formed in SCC25 and CAL‐27 cells in the sh‐hsa_circ_0005325 group was significantly lower. These findings indicate that inhibiting the expression of hsa_circ_0005325 suppressed lumen formation in SCC25 and CAL‐27 cells. By inhibiting the expression of hsa_circ_0005325 in OSCC tumor tissues, the expression of miR‐433‐3p in SCC25 and CAL‐27 cells was inhibited, and in another group, the expression of HMGA2 in SCC25 and CAL‐27 cells was promoted. Compared with that in the sh‐hsa_circ_0005325 group, the number of tubes formed in SCC25 and CAL‐27 cells in the sh‐hsa_circ_0005325+miR‐433‐3p inhibitor group was significantly greater. These findings indicate that inhibiting the expression of miR‐433‐3p promoted lumen formation in SCC25 and CAL‐27 cells. Compared with that in the sh‐hsa_circ_0005325 group, the number of tubes formed in SCC25 and CAL‐27 cells in the sh‐hsa_circ_0005325+LV‐HMGA2 group also increased significantly. These findings indicate that HMGA2 upregulates the expression of VEGFA, thereby promoting the lumen formation in SCC25 and CAL‐27 cells.

## 6 Conclusions

In summary, in this study, we found that circ_0005325 regulates the proliferation, apoptosis, colony formation, angiogenesis, and migration of OSCC cells by modulating the miR‐433‐3p/HMGA2 axis. Although some results were obtained in this study, the potential pathways of the Hsa_circ_0005325/miR‐433‐3p/HMGA2 axis in OSCC tumorigenesis still need to be further investigated.

## Author Contributions

**Zhihan Lin:** conceptualization, resources, data curation, software, formal analysis, validation, investigation, methodology, writing – original draft, writing – review and editing. **Yating Fu:** resources, data curation, investigation, methodology, writing – review and editing. **Lei Mao:** Conceptualization, formal analysis, supervision, methodology. **Hongjuan Yan:** resources, investigation, methodology. **Wen Liu:** Resources, data curation, formal analysis, supervision, visualization, writing – review and editing. **Xiaoxue Tang:** conceptualization, formal analysis, supervision, funding acquisition, methodology, project administration, writing – review and editing.

## Ethics Statement

Our study did not require further ethics committee approval as it did not involve animal or human clinical trials and was not unethical.

## Conflicts of Interest

The authors declare no conflicts of interest.

## Data Availability

Data supporting this study are available from the corresponding author upon request.
