# Correction to “Hsa_circ_0005325 Regulates the Proliferation, Apoptosis, Colony Formation, Migration, and Angiogenesis‐Promoting Behavior of Oral Squamous Cell Carcinoma Cells Through the miR‐433‐3p/HMGA2 AxisHsa_circ_0005325 Regulates OSCC via miR‐433‐3p/HMGA2 Axis”

**DOI:** 10.1002/cre2.70304

**Published:** 2026-02-10

Lin, Z., Y. Fu, L. Mao, et al. 2025. “Hsa_circ_0005325 Regulates the Proliferation, Apoptosis, Colony Formation, Migration, and Angiogenesis‐Promoting Behavior of Oral Squamous Cell Carcinoma Cells Through the miR‐433‐3p/HMGA2 Axis.” *Clinical and Experimental Dental Research*. 11, no. 5: e70208. doi:10.1002/cre2.70208

**1. Author Order Correction:**

Zhihan Lin (originally listed as the first author) and Yating Fu (originally listed as the second author) made equally significant contributions to the study. They jointly participated in core work including experimental design, data collection and analysis, and drafting of the original manuscript. In line with academic guidelines for co‐first authorship and with the consensus of all co‐authors, they should be designated as co‐first authors.

In page 1 of the “Author” section, the text “Zhihan Lin1 | Yating Fu2 | Lei Mao1 | Hongjuan Yan1 | Wen Liu3 | Xiaoxue Tang.” was incorrect. This should have read: “Zhihan Lin1# | Yating Fu2# | Lei Mao1 | Hongjuan Yan1 | Wen Liu3 | Xiaoxue Tang1.” #These authors contributed equally to this work and should be considered co‐first authors.

**2. Standardization of Author Affiliation:**

Due to the change in hospital name, the name of affiliation 1 has been changed accordingly.

In page 1 of the “Affiliation” section, the text “^1^Department of Stomatology, The First Affiliated Hospital, School of Medicine, Shihezi University, Shihezi, China | ^2^Department of Radiology of Urumqi Stomatological Hospital, Urumqi, Xinjiang, China | ^3^Department of Ultrasound, The First Affiliated Hospital of Shihezi University, Shihezi, China” was incorrect. This should have read: “^1^Department of Stomatology, The First Affiliated Hospital of Shihezi University, Shihezi, China | ^2^Department of Radiology of Urumqi Stomatological Hospital, Urumqi 830002, Xinjiang, China | ^3^Department of Ultrasound, The First Affiliated Hospital of Shihezi University, Shihezi, China”.

**3. Supplementation of Funding Information:**

Due to the addition of fund project support, the fund section needs to be modified.

In page 1 of the “Funding” section, the text “This study was supported by the National Natural Science Foundation of China (82160572) and Shihezi University Provides Independent Funding to Support University Level Scientific Research Projects (ZZZC2022065).” was incorrect. This should have read: “This study was supported by the National Natural Science Foundation of China (82160572) and Shihezi University Provides Independent Funding to Support University Level Scientific Research Projects (ZZZC2022065), and the 2025 Health Science and Technology Program of the XPCC (BTKY250072).”

We apologize for these errors.

